# Clustering Denoising of 2D LiDAR Scanning in Indoor Environment Based on Keyframe Extraction

**DOI:** 10.3390/s23010018

**Published:** 2022-12-20

**Authors:** Weiwei Hu, Keke Zhang, Lihuan Shao, Qinglei Lin, Yongzhu Hua, Jin Qin

**Affiliations:** 1 College of Electronics and Information, Hangzhou Dianzi University, Hangzhou 310018, China; 2 Hangzhou Pioneer Technology, Hangzhou 310018, China

**Keywords:** simulation localization and mapping (SLAM), LiDAR, clustering noise reduction, keyframe extraction

## Abstract

In the indoor laser simulation localization and mapping (SLAM) system, the signal emitted by the LiDAR sensor is easily affected by lights and objects with low reflectivity during the transmission process, resulting in more noise points in the laser scan. To solve the above problem, this paper proposes a clustering noise reduction method based on keyframe extraction. First, the dimension of a scan is reduced to a histogram, and the histogram is used to extract the keyframes. The scans that do not contain new environmental information are dropped. Secondly, the laser points in the keyframe are divided into different regions by the region segmentation method. Next, the points are separately clustered in different regions and it is attempted to merge the point sets from adjacent regions. This greatly reduces the dimension of clustering. Finally, the obtained clusters are filtered. The sets with the number of laser points lower than the threshold will be dropped as abnormal clusters. Different from the traditional clustering noise reduction method, the technique not only drops some unnecessary scans but also uses a region segmentation method to accelerate clustering. Therefore, it has better real-time performance and denoising effect. Experiments on the MIT dataset show that the method can improve the trajectory accuracy based on dropping a part of the scans and save a lot of time for the SLAM system. It is very friendly to mobile robots with limited computing resources.

## 1. Introduction

LiDAR is a kind of high-precision and high-stability sensor that is widely used in automatic driving [1], industrial measurement [2], unmanned aerial vehicles (UAV) [3], and other fields.

LiDAR sensors work by illuminating the target with a light pulse and measuring the characteristics of the return signal, where the target’s distance is obtained by calculating the round-trip delay of the reflected light. Although the technology has high accuracy, the pulse is sensitive to light, glass, and other factors. In this indoor environment, the laser points obtained by the sensor will inevitably be affected by noise. These noises can be mainly divided into three categories: isolated outliers, clustered outliers, and outliers near the signals. To obtain more accurate point cloud data, the original LiDAR scans need to be denoised.

The purpose of this paper is to denoise the LiDAR scans in a real-time simulation localization and mapping (SLAM) system with limited computing resources. Thus, this paper pays more attention to the real-time performance of the denoising methods. At present, the widely used traditional machine learning clustering methods include the Euclidean clustering method [4], K-means method [5], and density-based spatial clustering of applications with noise (DBSCAN) method [6,7]. K-means is mainly used to find circular or spherical clusters, which limits the clustering of spatial data. The Euclid clustering method uses a KD-tree to improve the computing speed, but this method is easy to cause nonlinear clustering of spatial data. DBSCAN method can cluster any geometric clusters with nonlinearity to a good level and is very robust to noise [8]. However, the time complexity of these methods is relatively high. The time complexity of the original DBSCAN method reaches $O\left(n^{2}*d\right)$, where *d* is the calculation time of the Euclidean distance, and this process often needs to be accelerated [6]. It is difficult for indoor mobile robots without strong computing power to run these methods in real-time.

**Figure 1:**
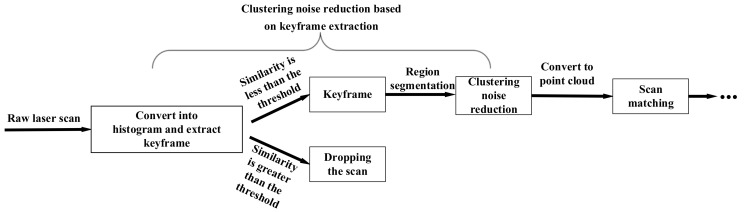
The process framework of the method and its position in the SLAM system. The method will be inserted into the SLAM system where it was before converting the 2D LiDAR data to the point cloud data.

At the same time, we notice that the moving speed of indoor mobile robots generally does not exceed 1 m/s. However, the scanning frequency of modern 2D LiDAR can generally reach 30 Hz or even 40 Hz. In other words, the robot captures a scan in 0.03 s. In this case, the environment represented by the adjacent scans does not change significantly. The mobile robot will capture and process many similar scans. In this case, if the traditional noise reduction method is adopted, all scans need to be processed. The processing of similar scans does not contribute significantly to updating the map and improving trajectory accuracy but rather increases the computational pressure on the system. Therefore, regarding the idea of reducing the number of scans for clustering noise reduction, this paper proposes a clustering noise reduction method based on keyframe extraction. We regard the latest scan as the target scan and the recent past keyframe as the reference scan. If the similarity between them is low, the target scan is considered to be a keyframe, which will be denoised and transmitted to the SLAM system. Otherwise, the scan will be considered a nonkeyframe and dropped. The extracted keyframe is also saved as a new reference scan. The structure of the clustering noise reduction method based on keyframe extraction proposed in the SLAM system is shown in Figure 1.

To improve the real-time performance of the keyframe extraction method, the scan dimension is reduced into a histogram, and the histogram is used to compare the similarity between scans. Meanwhile, to avoid dropping too many scans due to too high a similarity between adjacent scans, a sliding window is used to save multiple adjacent keyframes as reference scans. The target scan is compared with the reference scans in the window. If the similarity between the target scan and the reference scans is all relatively high, the target scan will be dropped. The keyframe extraction method will be inserted before the core function of the SLAM system. If the target scan is dropped, the scan will not participate in a series of subsequent steps such as noise reduction, scan matching, etc. It is mainly scan matching, which consumes a large number of computing resources [9]. In this way, for a nonkeyframe, the system only needs to spend less computing resources to calculate its similarity. It is beneficial to reduce the computational pressure of the system.

Next, in order to improve the clustering effect of the system, the region segmentation method is used in clustering processing, which is generally used when processing large-scale point clouds [10]. After the laser points in the scanning are divided into different regions using the region segmentation method, the laser points in the same region are clustered. At the same time, the angle difference method is used in the same area instead of solving the Euclidean distance. The Euclidean distance is only solved for the adjacent laser points in the adjacent area when the laser-point cloud blocks need to be merged. The Euclidean distance threshold will be adaptively adjusted according to the different regions. It can be seen from the analysis that the time complexity of the clustering noise reduction method is O(n∗a), and *a* is the time to calculate the angle difference between adjacent laser points in the region. Therefore, this method can improve the clustering effect while reducing its own time complexity. Finally, the obtained clusters are compared with the threshold. If the number of laser points in a cluster is less than the threshold, these laser points will be regarded as noise.

In general, the contributions of this paper are as follows:

1. A clustering noise reduction method based on keyframe extraction is proposed. Only the obtained keyframes are denoised. Since all scans without new environmental information are dropped, this method can not only effectively remove noise but also reduce the overall computational pressure of the system.
2. During the keyframe extraction, the dimension of a scan is reduced to a histogram, and the histogram is used for similarity comparison. The method reduces the influence of noise on similarity comparison to a certain extent. It can drop a portion of scans while preserving environmental characteristics.
3. In the process of clustering noise reduction, the region segmentation method is used. This method can reduce the dimension of clustering. It has a positive effect on reducing the time consumption of the clustering process and obtaining a better clustering effect. Meanwhile, it can improve the accuracy of LiDAR data to a certain extent.

The rest of this paper is organized as follows: Section 2 introduces the related work. Section 3 describes our proposed method in detail and presents the algorithm of the clustering noise reduction part. Section 4 presents the experimental results of this paper. Finally, conclusions and future work are discussed in Section 5.

## 2. Related Work

Since the keyframe extraction and clustering noise reduction in the method proposed are low couplings, this section will discuss the commonly used methods of keyframe extraction and noise reduction, respectively.

### 2.1. Keyframe Extraction

The keyframe extraction in this paper can be roughly divided into two steps. The first step is to reduce the dimension of the input LiDAR scans. The second step is to compare the similarity of the reduced dimension scans. Y. He et al. [11] chose to extract feature points from the scan to reduce the dimension of the input data. Although this method can achieve the purpose of dimension reduction well, it may consume a lot of time in the feature detection process. M. Boose et al. [12] used the method of constructing histograms to reduce the dimension of the input data. They convert the input scans into histograms. Compared with a LiDAR scan with thousands of points, the histogram has only a dozen columns, and the dimension of the data has been significantly reduced. In addition, the histogram is more flexible, and we can easily change parameters such as the number and size of the columns. However, M. Boose et al. used histograms as the core function of scan matching, which makes the matching accuracy excessively dependent on the accuracy of the histogram parameters. It is difficult to directly determine the parameters in the histogram in a SLAM system.

A widely used method to compare the similarity between scans is scan matching, such as iterative closest point (ICP) [13,14]. Although scan matching methods have higher accuracy, they tend to have higher time complexity [9], which does not meet the goal of reducing the computational pressure of the SLAM system in this paper. In [15], the histogram is regarded as a random variable with statistical characteristics, and the correlation of the histogram is calculated. In addition, there are also methods to solve the similarity between scans based on graph optimization theory [16,17], that is, to perform loop closure detection by comparing the similarity between scans. However, the essence of these methods is still scan matching.

### 2.2. Point Cloud Denoising

There are many related methods for the noise reduction of the point cloud. The method based on the voxel grid (VG) filter [18] is to define a corresponding 3D (2D) frame in the point cloud space to form a voxel grid. For each voxel grid, a point is selected to replace all the points in the grid. Since noise points are usually isolated outliers, such noise points can be removed using a voxel grid filter. The VG method is fast and simple to implement, but it is possible to remove normal points while removing noise points. Moreover, VG will down-sample the data points in the grid, and the environment information may be lost for the single-line LiDAR used indoors. Outliers are points in a scan that are significantly different from other data points and mainly represent noise in a scan. Based on this, some noise reduction methods based on outlier removal technology have been born, such as the radius outlier removal (ROR) [19] and statistical outlier removal (SOR) [20]. ROR is an easy-to-implement method that calculates the number of adjacent points of each point within its fixed search radius R. When the number of adjacent points is lower than the defined threshold, this point will be divided into an outlier. Since a fixed search radius is used, it is easy to mistake normal points as outliers when the distance between points is relatively far. Similar to ROR, SOR is also based on the information of adjacent points to remove outliers, but instead of using a fixed radius SOR distinguishes signals and noises by calculating the average distance between each point and its nearest K points. Although the performance of SOR is further improved compared to ROR, SOR seriously increases the computational overhead. Both the voxel-grid-based and outlier-based methods focus more on removing isolated outliers. In the area close to the sensor, noise points tend to cluster together, and the above methods often perform poorly in this case.

In order to cope with various situations of noise distribution, cluster-based noise reduction methods have been paid more and more attention. Clustering is an analytical method that uses distance or similarity between data to divide an entire dataset into groups. DBSCAN method is widely used in noise reduction [6]. It can cluster various shapes and effectively remove the clustered and isolated outliers. J. Kim et al. [8] proposed a graph-based spatial clustering algorithm. The algorithm uses the Delaunay triangulation tool commonly used in geometric spatial analysis and the concept of adjacent objects in DBSCAN to better segment point cloud while reducing clustering background noise. However, these methods are accompanied by high time complexity and often need to be accelerated. Running them in real-time is difficult for indoor mobile robots with limited computing resources.

In summary, the keyframe extraction method needs to be able to reduce the dimension of the input data, and the noise reduction method needs to be able to remove outliers in various distribution situations; both of them need to maintain a low time complexity to meet the real-time performance of mobile robots.

## 3. Clustering Noise Reduction Based on Keyframe Extraction

The keyframe extraction in the method proposed serves for clustering noise reduction, so this section introduces the keyframe extraction methods and clustering noise reduction methods used in turn.

### 3.1. Keyframe Extraction of 2D LiDAR

The basic idea of extracting keyframes is to compare the similarity of the target scan and the reference scan. If the similarity is high, the target scan will be dropped; otherwise, it will be regarded as a keyframe and saved as a new reference scan. In order to reduce the time complexity of calculating similarity, the method of constructing a histogram in [11] is used to process the 2D LiDAR data. In addition, to prevent dropping too many target scans, we use sliding windows to save a certain number of reference scans.

#### 3.1.1. Preprocessing

The raw 2D LiDAR data are generally stored in polar coordinates. Assuming that the point set composed of *N* laser points is *P*, we use polar coordinates (*R_i_*, *θ_i_*) to represent each point *P_i_* in the set *P*. The point set *P* can be expressed as Formula (1):


(1) $$P=\left\{p_{i}=\left(R_{i},\theta_{i}\right),\;i\in \left[1,\;N\right]\right\}.$$

In the actual environment, the raw LiDAR data will have some useless points beyond the range or meaningless laser points. They will make the construction of the histogram fail. Therefore, a simple distance filter needs to be designed to eliminate the meaningless and useless points: where min_*range* and max_*range* can be obtained directly in the scan. The processing flow of the distance filter is shown in Algorithm 1.
**Algorithm 1** Distance Filter**Input:** Laser point *p_i_* from *P* set**Output:** Filtered set *S* 1. **for**
*p_i_* ∈ *P* do 2. **if**
$R_{i}\ll \min\_\mathrm{range}||\;R_{i}\gg \max\_\mathrm{range}$
**then** 3. Drop the point 4. **else**
*S* ← *P_i_* 5. **end if**
 6.**end for**
 7.**return**
*S*

#### 3.1.2. Construct Histogram

In order to reduce the dimension of a frame of LiDAR data, this paper chooses the method of converting it into a two-dimensional histogram. At the same time, the histogram is regarded as a random variable with statistical characteristics [15], and the Pearson correlation coefficient [21] is used to calculate the similarity.

Two-dimensional LiDAR data are generally laser points sorted according to the angle value from small to large in the polar coordinate system. We can obtain each laser point’s distance and angle in the polar coordinate system. Obviously, we can construct the histogram by taking advantage of the characteristic that the angle value of the laser point increases regularly. For a frame of laser points, they are divided into several angle intervals according to the size of the angle, and the angle difference of each angle interval is equal. The different angle intervals are taken as the first dimension of the two-dimensional histogram. There are two ideas for the second dimension of the histogram: the first is to use the number of laser points in the angle interval, and the other is to use the average distance of laser points in the angle interval. Since the number of effective laser points between adjacent scans is relatively close, the number of laser points in each angle interval is also very close. Therefore, using the first idea will make the similarity between the target and the reference scans extremely high, and it will easily cause an excessive drop in the scan. Therefore, this paper uses the second idea. On the one hand, using the average distance of laser points can reduce the influence of noise on the construction of histograms. On the other hand, the average distance can also reflect the changes of the environmental characteristics.

#### 3.1.3. Calculate Similarity

Because histograms are regarded as random variables with statistical characteristics, and adjacent histograms are generally similar, it can be considered that histograms corresponding to different scans have the same distribution.

The Pearson correlation coefficient (PCC) was selected to calculate the similarity of histograms. PCC is a statistical indicator used to measure the strength and direction of the linear relationship between two random variables [21]. PCC is very sensitive to the fluctuation of values and can capture the small changes in variables, which is very helpful in solving the similarity of histograms. Most importantly, the time complexity of PCC under a sliding window is *O*(*n* ∗ *m*), where *n* is the number of columns in the histogram, and *m* is the number of reference scans in the window. It can be concluded that the low time complexity of PCC does not bring an obvious computational burden to the SLAM system. The PCC of the histogram *X*,*Y* is formula (2):

(2) $$P_{X,Y}=\frac{n\left(\sum_{i=1}^{n}x_{i}y_{i}\right)-(\sum_{i=1}^{n}x_{i})\left(\sum_{i=1}^{n}y_{i}\right)}{\sqrt{\left[n\sum_{i=1}^{n}x_{i}^{2}-{\left(\sum_{i=1}^{n}x_{i}\right)}^{2}\right]\left[n\sum_{i=1}^{n}y_{i}^{2}-{\left(\sum_{i=1}^{n}y_{i}\right)}^{2}\right]}},$$

where *n* is the number of columns of the histogram. *x_i_* and *y_i_* are the value of each column of the *X* and *Y* histograms, that is, the average value of the distance of the laser points in the interval. The value of *P_X,Y_* is between −1 and 1. When the value is −1, the two variables are negatively linearly correlated, 0 indicates no linear correlation, and 1 indicates positive linear correlation. The greater the absolute value of *P_X,Y_*, the stronger the correlation between the two variables. For the case of this paper, we can analyze that the value of *P_X,Y_* should be close to 1.

#### 3.1.4. Select Parameters

At present, the following four parameters need to be considered:

1. *n*—the number of columns of the histogram;
2. *m*—the size of the sliding window;
3. *P_pair_*—the similarity threshold between two scans;
4. *P_threshold_*—the similarity threshold for dropping target scan.

The first is the selection of the number *n* of histogram columns. It can be analyzed that *n* should be equal to the number of angle intervals in Section 3.1.2. For example, in the MIT dataset [22], there are about 1000 laser points in a frame of data, and if n is set to 20, each column contains an average of 50 points. For a frame of laser data, the larger *n* is, the fewer the laser points and the greater the number of details that are contained in each column. When the contrary is true, fewer details are contained in each column. Two extreme cases can be considered. In the first case, the value of *n* is set as the number of laser points. That is, a column is a laser point. At this time, the similarity between histograms is very sensitive to each laser point and even noise. The similarity will be greatly reduced, which cannot play the role of extracting keyframes. The second case is to set the value of *n* to 1, in which case all scans will become similar, causing too many scans to be dropped. Therefore, we need to select the appropriate *n* value. According to the experimental data given in Section 4, it is appropriate to divide 1000 laser points within the range of 3*π*/4 into 15 to 20 columns for the MIT dataset.

The other three parameters are closely related. The past keyframes saved in the sliding window are used as the reference scans, and the latest scan is used as the target scan. The total similarity *P_common_* of the target scan and the reference scans should be the product of the similarity *P_couple_* of each reference scan and the target scan. If *P_common_* is less than the similarity threshold *P_threshold_*, the target scan will be kept as a keyframe; otherwise, the scan will be dropped. The relational expressions of *P_couple_* and *P_common_*, and the relational expressions of *P_pair_* and *P_threshold_* are as follows in Formula (3):


(3) $$\begin{array}{l} P_{\mathrm{common}}=\overset{m}{\underset{i=1}{\prod }}P_{\mathrm{couple}}={\left(P_{\mathrm{couple}}\right)}^{m}\\ P_{\mathrm{threshold}}=\overset{m}{\underset{i=1}{\prod }}P_{\mathrm{pair}}={\left(P_{\mathrm{pair}}\right)}^{m}. \end{array}$$

According to Formula (3), it can be concluded that the smaller the value of *m*, the larger the value of *P_common_* and the easier it is to drop the target scan. Therefore, the value of *m* directly affects the selection of keyframes. Generally, if the moving speed *V_robot_* of the mobile robot increases, the obtained *P_couple_* will decrease. If the value of *m* remains unchanged at this time, the value of *P_common_* will also decrease. At this time, in order to ensure that enough scans can be dropped, it is necessary to appropriately reduce the value of *m*. According to this, we can find the approximate relationship between *m* and *V_robot_*: the larger *V_robot_* is, the smaller *m* needs to be. A more accurate description is that the window size *m* is directly related to the average distance (Δ*x*) that the robot passes between two scans. The definition of Δ*x* is shown in Formula (4):

(4) $$\Delta x=\frac{V_{\mathrm{robot}}}{f},$$

where *V_robot_* is in centimeters and *f* is the scanning frequency of the LiDAR. The quantitative relationship between Δ*x* and *m* is shown in Formula (5):


(5) $$18<m*\Delta x^{2}<30.$$

According to the experimental data given in Section 4, *f* is fixed at 40 Hz, *m* = 8 when *V_robot_* = 0.752 m/s, and *m* = 9 when *V_robot_* = 0.575 m/s.

**Figure 2:**
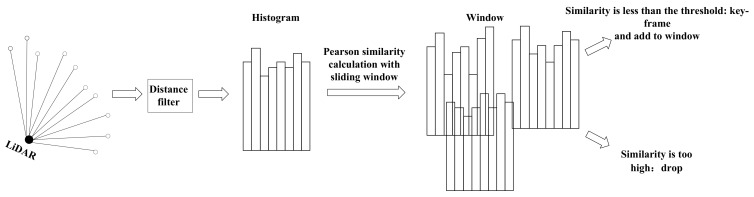
The process of keyframe extraction. Scans that meet the similarity threshold will be treated as keyframes and added to the window to update the window.

In conclusion, the process of keyframe extraction can be described in Figure 2.

### 3.2. Clustering Noise Reduction Method

#### 3.2.1. Region Segmentation

According to Section 3.1.1, the original two-dimensional LiDAR data is in the polar coordinate system. Because we are studying two-dimensional LiDAR, we do not consider the *z*-axis direction.

**Figure 3:**
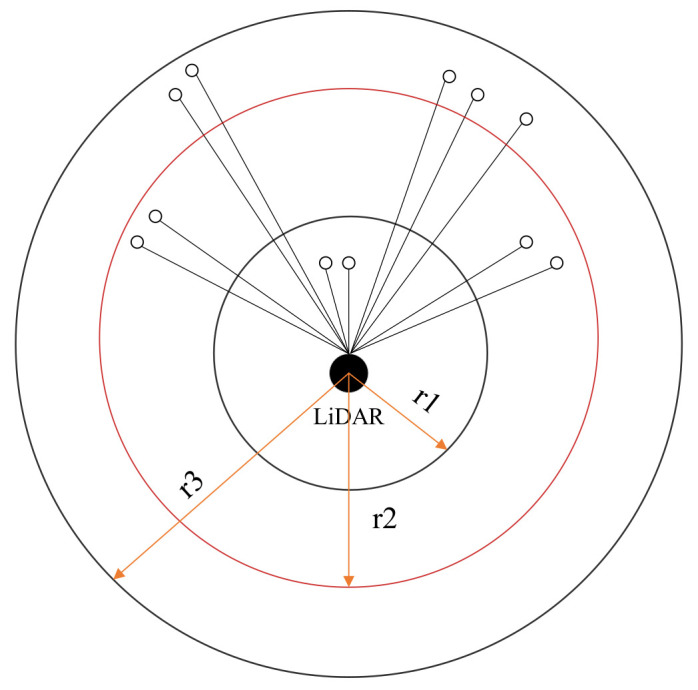
Region segmentation method. The LiDAR is located in the center of all regions, each ring has an equal area, and a ring represents a laser points region. There are three regions in the figure.

In the polar coordinate system, the regions are divided according to the principle of equal ring area of different regions. The specific division method is shown in Figure 3. The ring represents the region obtained after region segmentation. In order to ensure that the area of each ring is equal, the radius relationship of each circle should follow Equation (6):

(6) $$\pi r_{1}{}^{2}=\pi r_{2}{}^{2}-\pi r_{1}{}^{2}=\pi r_{3}{}^{2}-\pi r_{2}{}^{2}=\cdots =\pi r_{i}{}^{2}-\pi r_{i-1}{}^{2},$$

where *r_i_*(*i* = 1,2⋯*t*) represents the radius of the *i−th* circular area and *t* represents the number of regions. According to the experimental data in Section 4, *t* = 5. The Formula (6) can be simplified to Formula (7):

(7) $$r_{i}=\sqrt{i}r_{1},$$

where *r*_1_ can be obtained from the distance *r_max_* of the farthest laser point in the scan and *r_max_* can be obtained directly from the 2D LiDAR data. The relationship between *r*_1_ and *r_max_* is shown in Formula (8):


(8) $$r_{1}=\frac{r_{\max}}{\sqrt{t}}.$$

#### 3.2.2. Clustering

**Figure 4:**
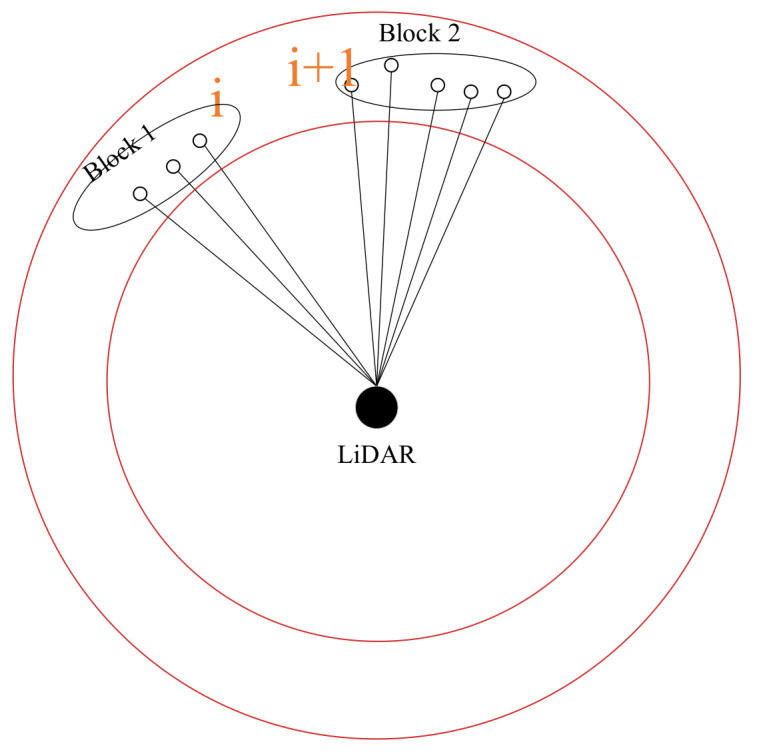
Clustering of laser points. The angle difference between points *i* and *i*+1 in the same region is greater than the *θ_threshold_*, and they are divided into different clusters.

After region segmentation, the method of calculating the angle difference between adjacent points in the same region is used for clustering. If the angle difference between two points is greater than the threshold *θ_threshold_*, it is considered to be the end of a cluster and the beginning of a new cluster. The detail is shown in Figure 4. At this time, different regional clusters do not affect each other.

Because it is a clustering for a certain region, the maximum distance in this region is fixed (greater than or equal to the maximum distance of laser points in this region), which is the radius of this circle. Similarly, the minimum distance in the region can also be fixed (less than the minimum distance of the laser points in this region), which is the radius of the previous circle. Since the maximum and minimum distances in a region are determined, it can be considered that the angle difference threshold and the Euclidean distance threshold have equivalent effects in the region. Moreover, the time complexity of calculating the angle difference between points is relatively low, which is beneficial to enhancing the real-time performance in the clustering process.

#### 3.2.3. Merge Laser-Point Cloud Blocks

Aiming at the problem that the same object may be divided into different regions and thus clustered into different clusters, a method of merging laser-point cloud blocks in adjacent regions is used in this paper. It should be noted that it is not necessary to judge whether the laser-point cloud blocks in nonadjacent regions can be merged. On the one hand, cross-region blocks are generally far away and cannot be merged. On the other hand, if cross-region blocks can be merged, there will be blocks that can be merged in the region between them.

We use the angle difference threshold (*θ_threshold_*) and Euclidean distance threshold (*d_threshold_*) to judge whether the two laser-point cloud blocks can be merged. The Euclidean distance threshold is judged only if the angle difference threshold is met. If the angle threshold is not met, it is directly considered that the two laser-point cloud blocks cannot be merged. We only calculate the two points with the closest angles of the two laser-point cloud blocks to judge whether the above thresholds can be met. Because the scan data are in the polar coordinate system, the Euclidean distance is calculated as in Formula (9):

(9) $$d_{i,i+1}=\sqrt{R_{i}^{2}+R_{i+1}^{2}-2R_{i}R_{i+1}\cos\Delta \theta },$$

where *R_i_* and *R*_*i*+1_ represent the measurement distances of the points *i* and *i* + 1. Δ*θ* denotes the angle difference of them. The distance threshold *d_threshold_* will change adaptively as the region changes. If the distance threshold between region *i* and *i* + 1 is calculated, the calculation method is as shown in Formula (10):

(10) $$d_{\mathrm{threshold}}=\sqrt{r_{i}^{2}+r_{i}^{2}-2r_{i}r_{i}\cos\theta_{\mathrm{threshold}}},$$

where *r_i_* represents the radius of the circle corresponding to the region *i*. According to the experimental data in Section 4, $\theta_{\mathrm{threshold}}=10*\theta_{\mathrm{resolution}}$, *θ_resolution_* is the angular resolution of the LiDAR.

**Figure 5:**
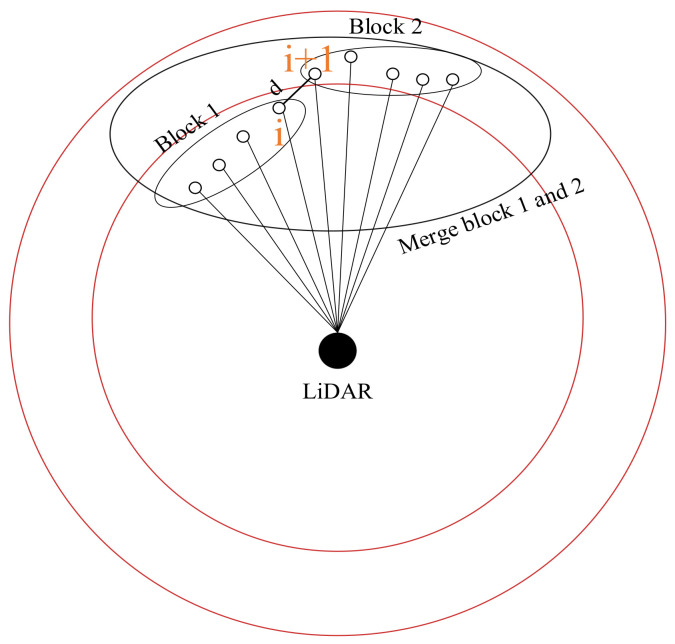
Merge laser-point cloud blocks. Calculate using points *i* and *i*+1, *d* is the distance between the two points. Blocks 1 and 2 satisfy both the angle threshold and distance threshold.

**Figure 6:**
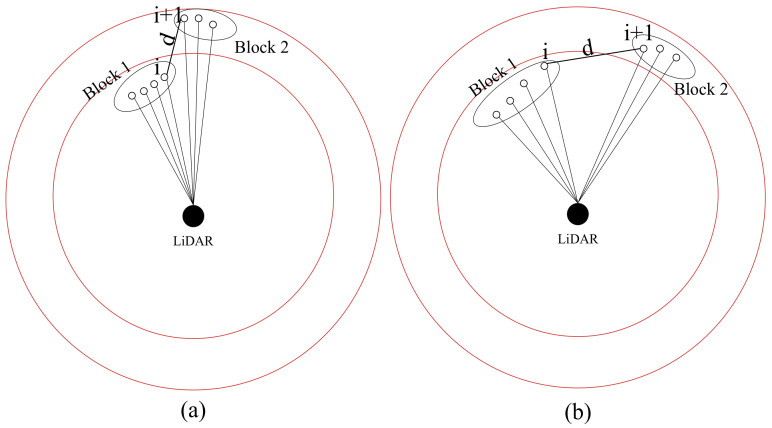
Laser-point cloud blocks cannot be merged. Calculate using points *i* and *i*+1, *d* is the distance between the two points. (**a**) The angle threshold is met, but the distance threshold is not. (**b**) The angle threshold is not met.

When two laser-point cloud blocks meet the angle threshold and the distance threshold at the same time, it is considered that the two blocks can be merged into one block, as shown in Figure 5. When the two laser-point clouds meet the angle threshold and do not meet the distance threshold, as shown in Figure 6a, the laser-point clouds do not meet the angle threshold, as shown in Figure 6b.

#### 3.2.4. Filtering of Clusters

We regard the obtained laser-point cloud blocks as clusters. According to the description in Section 3.2, we can use the Algorithm 2 representation for the cluster denoising method. According to the experimental data in Section 4, when the number of laser points in a cluster is less than *m_threshold_*, we consider it abnormal.
**Algorithm 2** Clustering Noise Reduction Method**Input:** Set *P* of laser points from keyframe.**Output:** Set *S* of laser points after clustering noise reduction processing. 1. The points are divided into *t* regions, and the points set is *T_j_*(*j* = 1,2…*t*). 2. Cluster the points in different regions. 3. **for**
*p_i_* ∈ *T_i_* && *j* = 1,2…*t*
**do** 4.  **if**
*θ*_*i*“1_ − *θ_i_* > *θ_threshold_*
**then** 5. *T_j,k_* is a block within region *j*. *θ_start_* is the starting point of *T_j,k_*. 6.  $T_{j,k}\leftarrow \left\{\theta_{\mathrm{start}}\ldots \theta_{i}\right\}$
 7.  *θ_start_* ← *θ*_*i*+1_ and *k* ++ 8.  **end if**
 9.
**end for**
 10. Merge Laser-point Cloud Blocks. *m* and *n* are indexes of blocks. 11. **for** $T_{j,m},T_{j+1,n}\;\&\&\;j=1,2\ldots t-1$ 12. $\left\{R_{i},\theta_{i}\right\}$ is the rightmost or leftmost point of block $T_{j,m}$. $\left\{R_{h},\theta_{h}\right\}$ is the leftmost or      rightmost point of block $T_{j+1,n}$. 13.  **if**
$\left|\theta_{i}-\theta_{h}\right|\ll \theta_{\mathrm{threshold}}\;\&\&\;d_{i,h}\ll d_{\mathrm{threshold}}$
**then** 14.  $T_{j,m}\leftarrow \left\{T_{j,m},T_{j+1,n}\right\}$
 15.  **end if**
 16.
**end for**
 17. *T_j,k_* is the final cluster. 18. **for** all *T_j,k_*
**do** 19.  **if**
$\mathrm{Number}\left(T_{j,k}\right)<m_{\mathrm{threshold}}$
**then** 20.  Drop *T_j,k_* 21.  **else**
$S\leftarrow T_{j,k}$ 22.  **end if**
 23.
**end for**
 24. **return**
*S*

## 4. Experiment

In order to verify the effectiveness and real-time performance of the proposed method, we chose to integrate it into Google’s Cartographer algorithm [23]. The MIT dataset [22] was selected for experimental testing because the MIT dataset contains a large amount of two-dimensional LiDAR data, and the official website of the dataset provides real trajectory files. All experiments were conducted on Ubuntu 18.04, ROS melodic, Intel Core i5-1035G1 @ 1 GHz, 8 GB RAM.

### 4.1. Experimental Setup

**Figure 7:**
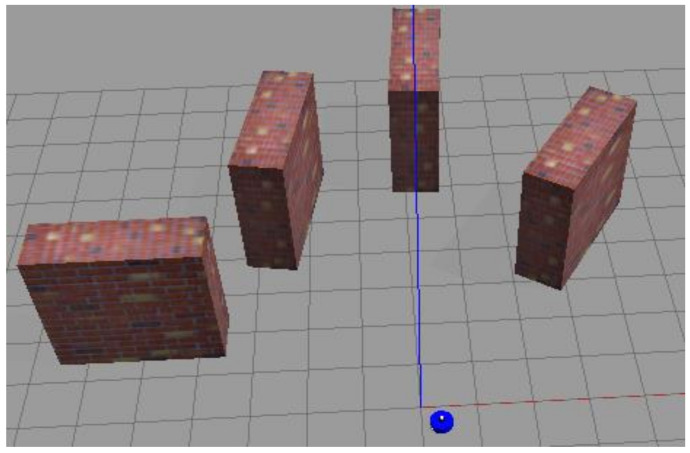
Three-dimensional simulation environment and mobile robot. Red rectangular blocks represent obstacles, and blue cylinder represents mobile robots.

In order to independently verify the feasibility and effectiveness of the clustering noise reduction method, in Section 4.2, we design a simulation experiment and built a virtual physical environment in Gazebo, as shown in Figure 7. The computing resources of indoor mobile robots are limited. One of the goals of this paper is to reduce the overall computational pressure of the system. To verify whether this goal is achieved, in Section 4.3, we use the MIT dataset to experiment and record the actual time consumption of the main steps. In order to further verify the effectiveness of the proposed method, in Section 4.4, we record the RMSE values of the trajectory under different conditions. In addition, our method is also compared with DBSCAN method.

### 4.2. Gazebo Simulation Experiment

Because the clustering noise reduction method is to cluster laser points and then judge whether they are abnormal points according to the number of laser points in the cluster, it is mainly necessary to verify the clustering effect. We set up a virtual physical environment using gazebo, as shown in Figure 7. In addition, a mobile robot equipped with a LiDAR sensor and a two-wheel differential drive module was also built. The range of the LiDAR sensor is 0.1–12 m, the scanning frequency is 10 Hz, the angular resolution is 0.5°, and the maximum number of sequence points obtained by scanning is 720.

**Figure 8:**
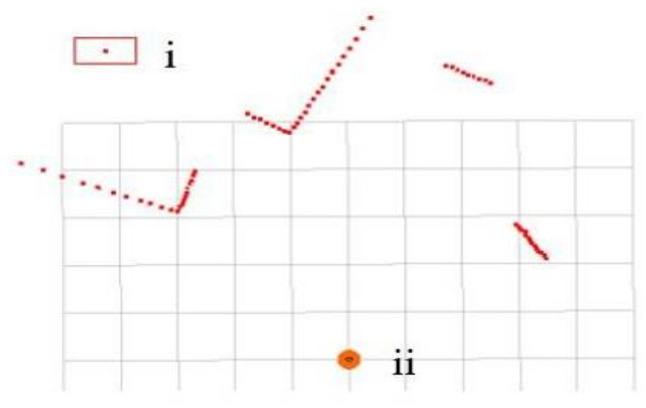
A frame of LiDAR scanning in the simulation environment. The points in the figure represent the point cloud of obstacles. (i) Indicates an abnormal laser point; (ii) refers to a robot. The side length of each grid is 1 m.

**Figure 9:**
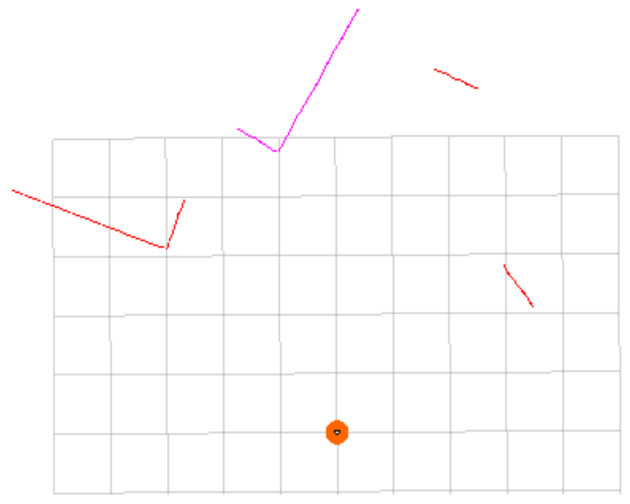
Clustering noise reduction results. The scan is clustered into 4 clusters, and the outlier is removed. Different lines represent different clusters. The color of lines has no special meaning, just to distinguish different clusters. Visualization using the MarkerArray plugin of RVIZ.

In this environment, the original LiDAR scanning of one frame is shown in Figure 8. The scan in Figure 8 is subjected to clustering noise reduction processing, and the parameters are selected as *t* = 5, *θ_threshold_* = 10 ∗ *θ_resolution_*, and *m_threshold_* = 10. The clustering situation is shown in Figure 9. It can be seen from Figure 9 that four clusters were finally obtained after clustering. Each cluster is represented by a line, and the abnormal point in Figure 8 is eliminated. It indicates that the clustering noise reduction method is effective.

### 4.3. Actual Time Consumption

If the method proposed in this paper is inserted into the SLAM system according to the process shown in Figure 1, it will not contribute to the real-time performance of the mobile robot, and then the method will be meaningless.

**Figure 10:**
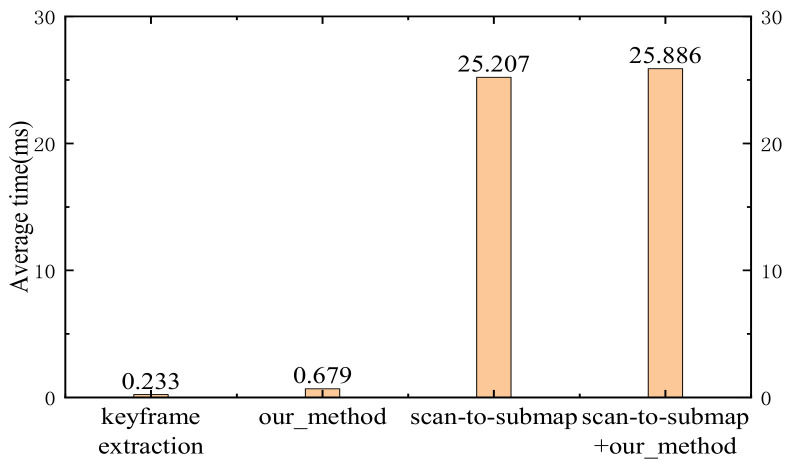
In the Cartographer algorithm, the time consumed comparison between the keyframe extraction method, our method, and the scan-to-submap method in processing a frame of data.

This experiment uses the 2012-01-18-09-09-07.bag package in the MIT dataset. The scan-to-submap method was used in the scan matching part of the Cartographer algorithm. Therefore, we need to record the average time consumption of scan-to-submap, keyframe extraction method, and the proposed method, respectively. The result is shown in Figure 10. The Cartographer algorithm is a graph-based SLAM system that can be roughly divided into two parts: front-end scan matching and back-end pose graph optimization. In the original Cartographer algorithm, all scans must go through the scan-to-submap step and then be inserted into the pose graph for optimization. At this time, it takes about 25.207 ms to process a frame scan only in the front-end of the system. After the proposed method is inserted into the Cartographer algorithm in the way shown in Figure 10, the keyframes will undergo noise reduction, scan-to-submap, and other operations, while the nonkeyframes will be dropped. At this time, for a frame of the scan, if the similarity is high after keyframe extraction, it will be dropped, so it will only take about 0.233 ms, instead of the original 25.207 ms, which is only 0.92% of the original time spent. If the similarity is low, it will take about 25.886 ms, which is 102.69% of the original time spent. The increase in time spent is not obvious. More importantly, according to Section 4.4, the proposed method can drop nearly 50% of scans. It can be roughly concluded that the Cartographer algorithm inserted with the proposed method can save about 48% of the time only in its front-end part. The proposed method can save more time if the time consumed by scans in the back-end optimization is considered. Therefore, the proposed method can reduce the overall computational pressure of the system.

### 4.4. Dataset Experiment

**Figure 11:**
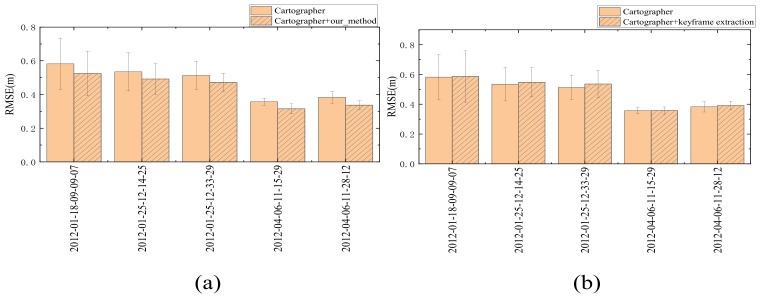
Columnar error diagram of two usage modes. (**a**,**b**) represent the influence of different methods on the trajectory accuracy. Figure (**a**) shows that our method can improve the trajectory accuracy to a certain extent. Figure (**b**) shows that the keyframe extraction method does not reduce the trajectory accuracy.

In order to verify the effectiveness of the proposed method, we chose to use the Cartographer algorithm on the MIT dataset to test the proposed method. In order to ensure that the selected keyframe extraction parameters are effective, this paper also gives the RMSE value of the trajectory when only the keyframe extraction method is used. The specific experimental results on the MIT dataset are shown in Table 1. The more intuitive results of the change of RMSE values for the two usage methods are shown in Figure 11. According to Table 1 and Figure 11, it can be seen that within the allowable error range, after selecting appropriate parameters, the trajectory accuracy of only using the keyframe extraction method is the same as that of the original trajectory accuracy. At the same time, about 50% of the scans can be dropped. We can also conclude that the algorithm’s accuracy of the trajectory generated by the algorithm is improved by about 10% after using the proposed method. It can be considered that the improvement of trajectory accuracy is due to the improvement of the accuracy of LiDAR data. This practically proves the effectiveness of the proposed method.

According to Section 3.1.4, the relationship between the parameter selection of key-frame extraction method and the moving speed of the robot follows Formulas (4) and (5). Therefore, we classify different bags according to the moving speed of the robot in Table 1. The moving speed *V_robot_* of the first three bags is about 0.752 m/s, and *V_robot_* of the last two bags is about 0.575 m/s. The *f* of the five bags is 40 Hz. According to the analysis, the larger *V_robot_* is, the lower the similarity between two consecutive frames of scans will be. In order to drop enough scans, it is necessary to reduce the sliding window size *m* and the threshold *P_pair_* at the same time. Because the value of *P_pair_* is reduced and, according to Formula (2), there is a direct relationship between *P_pair_* and *n*, the reduction of *P_pair_* indicates that the histogram can better reflect the details and changes of the scan, so it is necessary to increase the value of *n*. Based on the above analysis, the parameters selected for the first three bags are *m* = 8, *n* = 20, *P_pair_* = 0.985, and *P_threshold_* = 0.88. The parameters selected by the last two bags are *m* = 9, *n* = 15, *P_pair_* = 0.99, and *P_threshold_* = 0.91, where *P_threshold_* is calculated according to Formula (3). For the clustering noise reduction part, because the five bags are all carried out indoors, the same parameters are selected in this paper, that is, *t* = 5, *θ_threshold_* = 10 ∗ *θ_resolution_*, and *m_threshold_* = 10.

**Figure 12:**
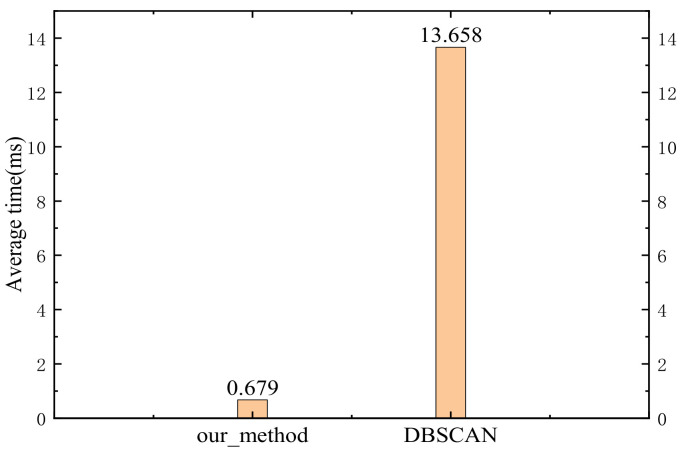
Comparison of the average time required by the proposed method and DBSCAN method in processing a frame of data.

In order to further illustrate the advantages of the proposed method in saving time and improving trajectory accuracy, we compared it with the DBSCAN method. We also inserted the DBSCAN method into the Cartographer algorithm, as shown in Figure 1. We also used the 2012-01-18-09-09-07.bag package to obtain the average time required by the DBSCAN method to process a frame of data. The result is shown in Figure 12. For this experiment, the DBSCAN method sets Eps to 0.7 m and minPts to 10. Eps represents the radius of the cluster, and minPts represents the threshold of the number of laser points in each cluster. If the number of laser points in a set with a radius of Eps is less than minPts, the cluster will be considered as a noise cluster.

In order to compare the effectiveness of the proposed method and DBSCAN in improving the trajectory accuracy, we used the DBSCAN method to carry out experiments in the above five bags. For each bag, we took *Eps* and *minPts* to the same values as in the above experiment. The specific experimental data are shown in Table 2.

According to Figure 12 and Table 2, the proposed method is better than the DBSCAN in saving computing resources and improving trajectory accuracy. Combined with the experimental result in Section 4.3, it can be concluded that the proposed method can improve the system’s trajectory accuracy while reducing the system’s overall computational pressure.

## 5. Conclusions

This paper proposes a clustering denoising method based on keyframe extraction. Since the separate clustering noise reduction method will bring additional computational pressure to the system, the keyframe extraction method was used to save computing resources. Only the extracted keyframes were used for noise reduction processing. According to the experimental results of the Cartographer algorithm on the MIT dataset, the proposed method can improve the system’s trajectory accuracy while reducing the system’s overall computational pressure.

First, in the keyframe extraction part, the scan dimension was reduced by constructing a histogram, and the PCC calculation method was used to calculate the similarity. To avoid dropping too many scans, a sliding window approach was used. According to the experiments on the MIT dataset in Section 4, it can be seen that, on the premise of ensuring the trajectory accuracy, part of and even half of the scans may be removed. It saves a lot of computational resources for the subsequent clustering noise reduction processing.

Secondly, for the clustering noise reduction part, the dimension in clustering was reduced by region segmentation, and calculating the Euclidean distance was replaced by calculating the angle difference. This method can obviously reduce the time complexity of the clustering process. According to the experiments in Section 4, the method has good real-time performance and can improve the accuracy of LiDAR data.

In a long corridor environment, all scans obtained were very similar. Since the proposed method drops all similar scans, it performed poorly in long corridor environments. In future work, we hope to find a simple corridor detection method as the “switch” of the keyframe extraction part and a more accurate quantitative relationship between the robot’s moving speed and the sliding window’s size. At the same time, we tried to use the idea of region segmentation to process 3D LiDAR data.

## Figures and Tables

**Table 1:** RMSE values and percentage of dropped scans for Cartographer on MIT dataset.

Bag	Cartographer	Cartographer + Keyframe-Extraction (m)	Dropped (%)	Cartographer + Our_Method (m)	Improvement (%)
2012-01-18-09-09-07	0.582 ± 0.153	0.587 ± 0.174	52	0.525 ± 0.132	10
2012-01-25-12-14-25	0.535 ± 0.113	0.548 ± 0.098	50	0.492 ± 0.092	8
2012-01-25-12-33-29	0.513 ± 0.083	0.536 ± 0.091	55	0.472 ± 0.054	8
2012-04-06-11-15-29	0.357 ± 0.021	0.358 ± 0.024	41	0.315 ± 0.030	12
2012-04-06-11-28-12	0.383 ± 0.035	0.392 ± 0.028	45	0.337 ± 0.026	12

**Table 2:** Comparison of the impact of our method and DBSCAN method on RMSE (m).

Bag	Cartographer	Cartographer + Our_Method	Cartographer + DBSCAN
2012-01-18-09-09-07	0.582 ± 0.153	0.525 ± 0.132	0.553 ± 0.143
2012-01-25-12-14-25	0.535 ± 0.113	0.492 ± 0.092	0.503 ± 0.088
2012-01-25-12-33-29	0.513 ± 0.083	0.472 ± 0.054	0.477 ± 0.065
2012-04-06-11-15-29	0.357 ± 0.021	0.315 ± 0.030	0.332 ± 0.025
2012-04-06-11-28-12	0.383 ± 0.035	0.337 ± 0.026	0.352 ± 0.028

## Data Availability

The datasets used and/or analyzed during the current study are available from the corresponding author on reasonable request.
